# Role of surfactant protein C in neonatal genetic disorders of the surfactant system: A case report

**DOI:** 10.1097/MD.0000000000028201

**Published:** 2021-12-17

**Authors:** Ya-Xin Tan, Shu-Jun Li, Hai-Tao Li, Xiao-Juan Yin, Bo Cheng, Jing-Li Guo, Na Li, Cheng-Zhong Zheng, Hong-Yu Chang

**Affiliations:** a Department of Pediatrics, the People's Liberation Army Rocket Force Characteristic Medical Center, Beijing, China; b Department of Pediatrics, the First Affiliated Hospital of Xinxiang Medical University, Xinxiang, Henan, China; c Department of Dermatology, the Seventh Medical Center of People's Liberation Army General Hospital, Beijing, China; d Bayi Children's Hospital, the Seven Medical Center of People's Liberation Army General Hospital, Beijing, China; e Department of Pediatrics, Hainan Hospital of the People's Liberation Army General Hospital, Sanya, Hainan Province, China; f The Second School of Clinical Medicine, Southern Medical University, Guangzhou, China; g The People's Liberation Army Rocket Force Characteristic Medical Center, Beijing, China; h Department of Pediatrics, the Strategic Support Force Medical Center of People's Liberation Army , Beijing, China.

**Keywords:** gene mutation, newborn, pulmonary surfactant, respiratory distress syndrome, surfactant protein C gene

## Abstract

**Rationale:**

Respiratory distress syndrome (RDS) refers to the symptoms of progressive dyspnea and respiratory failure in newborns shortly after birth. The clinical and genetic characteristics of patients with neonatal RDS have not been extensively reported.

**Patient concerns:**

A infant was in critical condition with repeated paroxysmal blood oxygen decline. Oxygen inhalation and noninvasive ventilator-assisted breathing relief were not effective. The etiology was unclear, and there was no family history of lung disease. Surface-active substance replacement therapy and positive pressure-assisted ventilation support were ineffective.

**Diagnosis:**

The infant was clinically diagnosed with RDS. Genetic tests revealed a heterozygous missense mutation in the c.168 surfactant protein C (SFTPC) gene.

**Interventions:**

Tracheal intubation was performed with invasive ventilator-assisted breathing, pulmonary surfactant was administered. Supportive treatment for liver protection and administration of a cardiotonic diuretic, vasodilator, human immunoglobulin (intravenous infusion), fresh frozen plasma, and suspended red blood cells were performed.

**Outcomes:**

The infant showed poor responses to respiratory and circulatory support, antibiotic treatment, and other treatment methods. The patient was discharged from hospital against the advice of us, cut off from us. The long-term prognosis of the patient after discharge remains unknown.

**Lessons:**

SFTPC gene mutations may be an important risk factor for the development of common lung diseases. Because of the important roles of surfactant functions and metabolism, mutations in these genes can affect the production and function of pulmonary surfactant, leading to severe lung disease in term newborns.

## 1 Introduction

Respiratory distress syndrome (RDS) refers to the symptoms of progressive dyspnea and respiratory failure in newborns shortly after birth, mainly due to deficiencies in pulmonary surfactant owing to immaturity.^[1,2]^ Clinical manifestations of progressive dyspnea, groaning, cyanosis, and inspiratory defects occur shortly after birth, and respiratory failure can subsequently appear in severe cases.^[3]^ The incidence of RDS is related to gestational age; lower gestational age is associated with a higher incidence of RDS, and lower birth weight is also associated with higher mortality.^[4]^

According to clinical analysis and epidemiological investigation, the incidence of RDS in China is about 1%.^[5]^ RDS is a common disease that threatens the life of the newborn seriously, and the incidence of term children has an obvious upward trend. In term infant, there are many unexplained and urgent RDS occurs. Respiratory support for more than a week and repeated use of PS does not significantly improve the condition, which bring some obstacles to clinical work. Besides, the pathogenesis of full-term RDS may become increasingly important with the study of molecular biology.

Surfactant protein B, surfactant protein C (SP-C), and ATP-binding cassette 3 (ABCA3) are important components of pulmonary surfactants.^[6]^ Although pulmonary surfactant proteins account for a small proportion of surfactant composition, they play important roles in surfactant homeostasis.^[7,8]^ Additionally, pathogenic variants in genes encoding surfactant protein B and ABCA3, transmitted by recessive traits in newborns, can cause severe, fatal lung disease.^[9]^

Although these variants encoding surfactant protein genes may be important risk factors for preterm infants with severe RDS, little is known about the relevance of these genetic variants. In this report, we describe a case of severe RDS in a term infant with mutations in the SFTPC gene.

## 2 Case description

### 2.1 Patient presentation and laboratory analyses

A term male infant (39 + 3 weeks of gestation, 3540 g birth weight) delivered by cesarean section experienced RDS at our institution. The infant had no history of intrauterine distress or birth asphyxia, no abnormal umbilical cord or placenta, and clear amniotic fluid. The Apgar scores were 9 at 1 minutes after birth and 10 at 5 to 10 minutes after birth. The infant appeared blue and purple when crying soon after birth, and the condition was not alleviated after providing hood oxygen. There was no relevant family history; the infant's parents were healthy, the marriage was not consanguineous, and there was no history of hereditary diseases. Additionally, the patient's sister had no similar medical history, and the mother, who had regular examinations during pregnancy, had no history of gestational diabetes mellitus or gestational hypertension.

Upon admission, the patient's temperature was 36.5°C, and the patient showed poor reactions and shortness of breath. There was no yellowing of the skin and no bleeding. The anterior fontanelle was soft. No abnormal secretion was observed in the nasal cavity. The lips showed some evidence of purpura. Evaluation of the lungs revealed thickness, with dry and wet vocal sounds. Heart sounds were normal, and the heart rate remained within the normal range, with a regular rhythm. The abdomen was soft, and the liver and spleen were not palpable. There were no abnormalities in bowel sounds. The limbs showed evidence of purpura, muscle tension was low, and reflexes were abnormal.

Laboratory analyses revealed the following routine blood test results: C-reactive protein, 6.0 mg/L; white blood cell count, 26.23 × 10^9^/L; red blood cell count, 4.6 × 10^12^/L; hemoglobin, 140 g/L; hematocrit, 41.6%; platelets, 421 × 10^9^/L; neutrophils, 80.7%; and lymphocyte percentage, 11.8%. The infection index was high. Biochemistry tests showed the following results: alanine aminotransferase, 42.1 U/L; total protein, 61.2 g/L; albumin, 37.8 g/L; total bilirubin, 9.84 μM; creatinine, 18.42 μM; uric acid, 149.0 μM; creatine kinase, 24.87 U/L; MB, 13.00 U/L. Computed tomography results showed that the pulmonary alveoli were diffusely patchy, and the ventilation bronchus could be observed. Chest radiographs showed an excessive reduction in double lung penetration, and the lung texture was blurred. Echocardiography revealed that the oval foramen was not closed. Abdominal ultrasonography showed no abnormalities.

The infant was clinically diagnosed with RDS and was suspected to have genetic surfactant dysfunction. Therefore, we performed next-generation sequencing to analyze genetic mutations. Briefly, 2 mL peripheral blood was collected from the infant and his family members to isolate genomic DNA using a QIAamp DNABlood Mini kit (Qiagen, Hilden, Germany). A targeted gene panel (Twist Bioscience, San Francisco, CA, USA) was designed for sequencing. The gene panel contained many candidate genes associated with hypoxic respiratory failure, such as *SFTPC*. Molecular analysis of these genes was performed by Beijing Deyi Oriental Translational Medicine Research Center Co., Ltd. according to standard laboratory procedures, and targeted gene capture and library sequencing were performed using an Illumina HiSeq2500 Analyzer (Illumina, San Diego, CA, USA) according to the manufacturer's protocol. Mutations were validated by Sanger sequencing, and relevant mutation data were evaluated based on the 1000 Genomes database and the National Coalition Building Institute dbSNP database (https://www.ncbi.nlm.nih.gov/variation/tools/1000genomes genome/). The possible effects of amino acid substitutions on protein structure and function were predicted using Sorting Intolerant From Tolerant, Protein Variation Effect Analyzer (Provean; http://provean.jcvi.org/protein_batch_submit.php?species=human), and Polymorphism Phenotyping 2 (PolyPhen-2; http://genetics.bwh.harvard.edu/pph2/).

This study was approved by the ethics committee of the People's Liberation Army Rocket Force Characteristic Medical Center. Clinical data were collected, and informed consent was obtained from the parents of the newborn.

## 3 Diagnostic assessment

The infant was admitted to the hospital in critical condition with repeated paroxysmal blood oxygen decline. Oxygen inhalation and noninvasive ventilator-assisted breathing relief were not effective; tracheal intubation was performed with invasive ventilator-assisted breathing, and multiple intratracheal drops of Guersu (pulmonary surfactant) were administered. Supportive treatment for liver protection and administration of a cardiotonic diuretic, vasodilator, human immunoglobulin (intravenous infusion), fresh frozen plasma, and suspended red blood cells were performed. The infant's condition continued to decline, and a ventilator was required to assist with breathing. Owing to poor swallowing function, the infant required tube feeding of milk, resulting in poor weight gain. With the support of tracheal intubation and invasive ventilator-assisted respiration, oxygen saturation was maintained at approximately 90%, and heart rate fluctuation based on ECG monitoring was 130 to 150 bpm. During hospitalization, withdrawal of the ventilator and oxygen was attempted many times; however, this was unsuccessful, and the patient showed poor responses to respiratory and circulatory support, antibiotic treatment, and other treatment methods. The family asked for the patient to be discharged from the hospital, and contrary to our recommendations, the patient was discharged. The long-term prognosis of the patient after discharge remains unknown.

**Figure 1:**
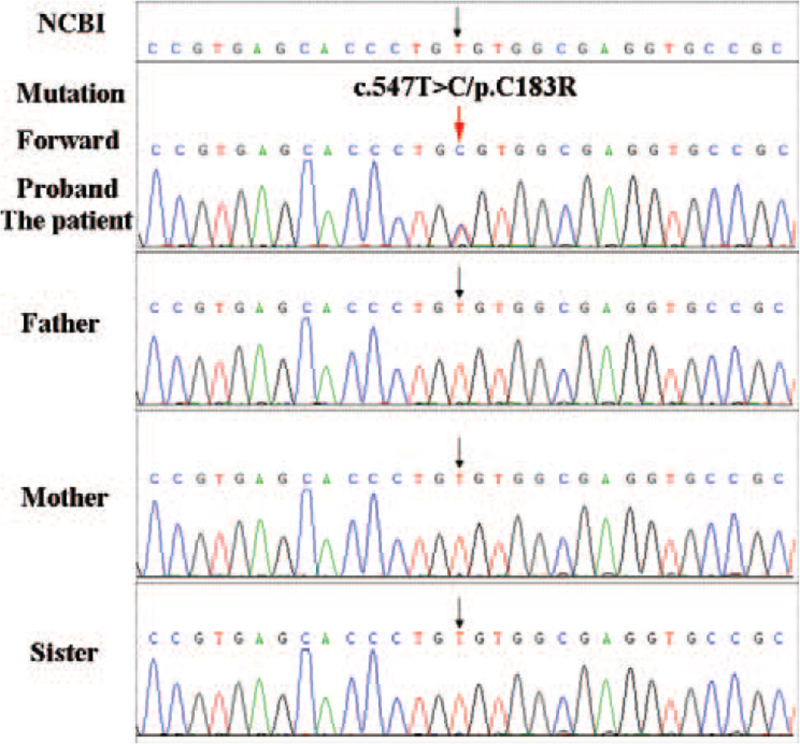
Genetic analysis of the family of the newborn with RDS. Sanger DNA sequencing chromatograms demonstrated heterozygosity for the mutation (c.547T > C) in the *SFTPC* gene. In the affected patient, a heterozygous T-to-C transition was observed at c.547 in exon 5, leading to p.C183R (red arrow). No family members or normal control sequences in National Center for Biotechnology Information (NCBI) (https://www.ncbi.nlm.nih.gov/protein/NP_001165828.1; black arrows) contained this mutation.

**Figure 2:**
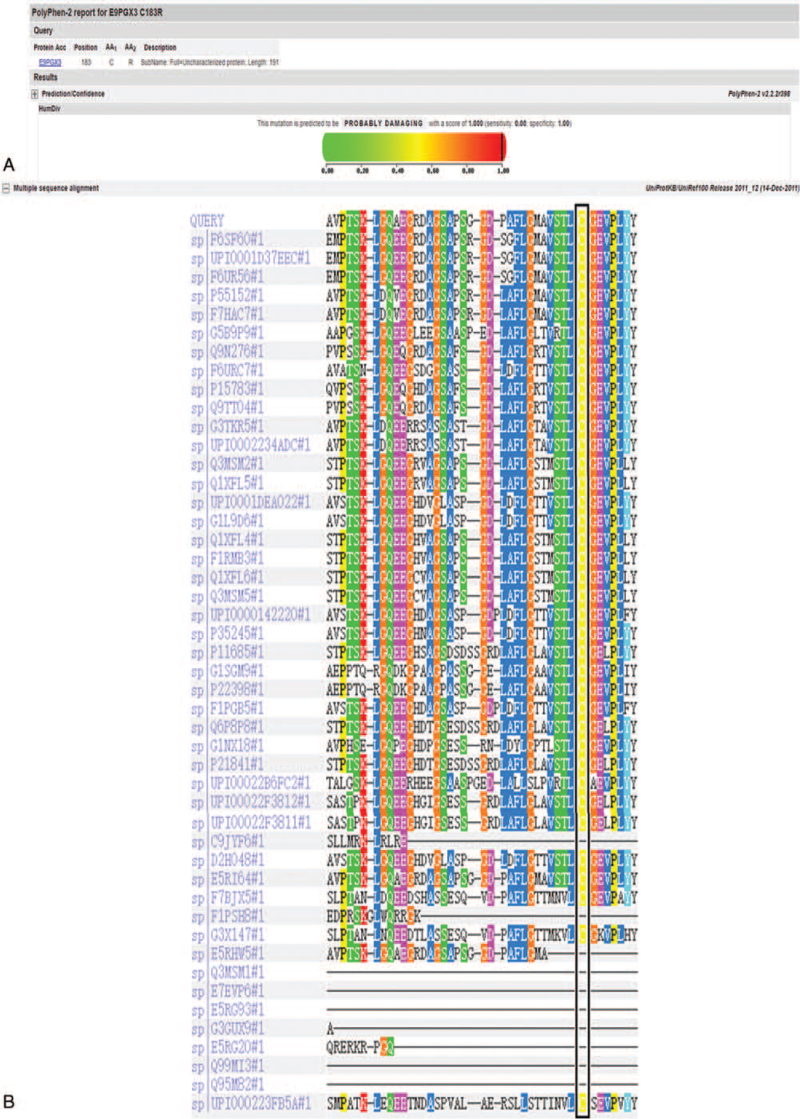
The amino acid substitution p.C183R in SFTPC protein was probably deleterious, as predicted by Polyphen-2 (A). Multiple sequenced alignment of the SFTPC protein indicated that the amino acid residue at position 183 was highly conserved across species (B).

**Figure 3:**
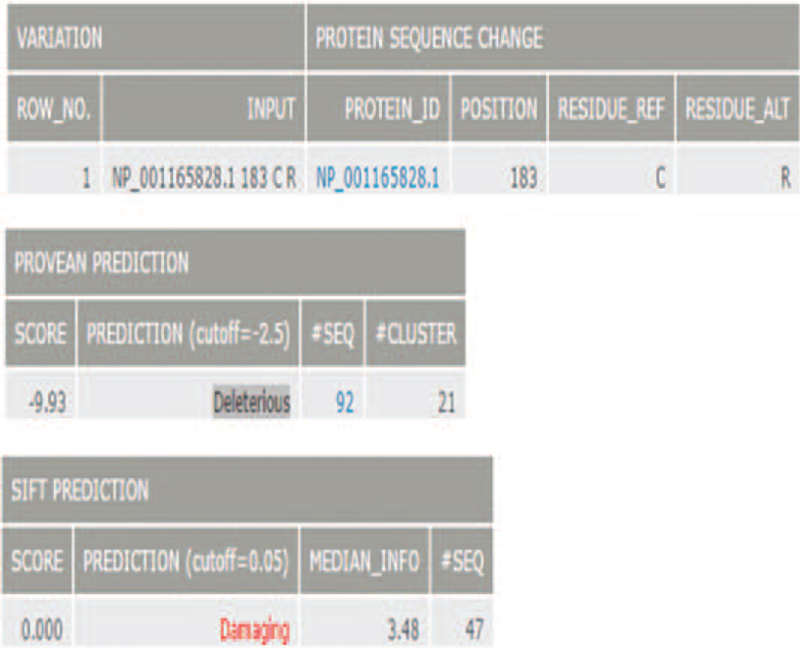
The amino acid substitution p.C183R in SFTPC protein was probably damaging, as predicted by Provean and SIFT.

From our next-generation sequencing analysis, a heterozygous mutation c.547T> C (p.C183R) in exon 5 of the *SFTPC* gene was identified in the infant. This mutation was not identified in any of the unaffected family members or in normal DNA sequences in the National Coalition Building Institute database (Fig. 1). The amino acid substitution p.C183R in SFTPC protein was likely to be deleterious, as predicted by Polyphen-2 (Fig. 2A). Multiple sequence alignment of the *SFTPC* gene indicated that the amino acid residue at this position (C183R) was highly conserved across species (Fig. 2B). Moreover, the amino acid substitution at position 183 in SFTPC was predicted to be “damaging” by sorting intolerant from tolerant (scores > 0.05) as well as “deleterious” by Provean analysis (Fig. 3).

## 4 Discussion

Defects in the SFTPC gene were first reported by Nogee et al in 2001.^[10]^ SFTPC is located on chromosome 8p21 and encodes SP-C, a hydrophobic 35-amino acid polypeptide secreted into the alveolar space by alveolar type II epithelial cells to facilitate reductions in surface tension.^[11]^ SP-C mutations are thought to be the cause of various lung diseases in patients of different ages. The typical presentation of SP-C mutations include dyspnea, cough, or wheezing with an onset between 2 and 12 months of age, as well as gradual cyanosis and failure to thrive.^[12–14]^ Additionally, children with SFTPB mutations have acute onset and severe phenotypes,^[15]^ and ABCA3 mutation phenotype is more variable and lethal.^[16]^ The presentation of SP-C dysfunction varies widely both in terms of the time of onset and the disease severity.^[17]^

Surfactant-related genes have been widely used in clinical practice as routine diagnostic items. With the development of improved sequencing technologies, all pathogenic genes can be sequenced under high-throughput conditions. In this study, we aimed to provide insights into the diagnosis of RDS based on genetic screening. Moreover, there are no effective treatments for metabolic defects in pulmonary surfactants, and most approaches involve symptomatic support therapy to alleviate symptoms and delay the disease process. Thus, identification of the genetic etiology of RDS may contribute to the development of novel therapies.

SFTPC gene defects exhibit autosomal dominant inheritance, and many studies have reported sporadic cases caused by new mutations. Studies in various countries around the world have shown that nearly 80% of children with SFTPC mutations have no family history of lung disease, and 40% to 80% of identified mutations are novel mutations, suggesting that this gene may be a mutational hotspot.^[18–20]^

Mutations found on the SFTPC gene can be classified into BRICHOS mutations and non-BRICHOS mutations depending on the sequence location. Functional studies show that BRICHOS mutations mainly lead to misfolding and abnormal processing of SP-C precursor proteins, leading to endoplasmic reticulum stress, while non-BRICHOS mutations affect the directed transport of SP-C protein within the cell.^[21,22]^ In our study, mutation including c.547T > C existed in SP-C gene was discovered in RDS infant. Meanwhile, the mutation above had not been reported before. We have a hypothetical that the mechanism might as following, the mutation in the SFTPC gene change amino acid sequence of SP-C protein, the accumulation of misfolding of SP-C precursor proteins (Pro SP-C), which leads to the accumulation of abnormal proteins, endoplasmic reticulum stress leading to the activation of apoptosis and release of pro-inflammatory cytokines.^[10,22]^

Our case highlights that RDS with SP-C dysfunction. SP-C dysfunction is an autosomal dominant disorder caused by an SFTPC mutation. A large part of the literature has reported sporadic cases caused by new mutations. Lot of studies show that the SFTPC mutation is a hot area of new mutations.^[23]^ Differences in the clinical manifestations of RDS may be related to differences in sex, race, genetic background, and environmental factors. However, for the SFTPC mutation c.547T > C (exon 5) identified in this study, no lung phenotypes have been reported in the literature worldwide. Thus, further studies are needed to identify the specific roles of this mutation site in the pulmonary phenotype based on omics-level analyses. Moreover, in our study, genetic analysis of the parents revealed no family history of this mutation. Accordingly, this mutation may be a novel mutation. In summary, we identified a previously undescribed mutation in the SFTPC gene and predicted the deleterious effects of this mutation on the structure and function of SFTPC protein. Because of the important roles of surfactant function and metabolism, mutations in this gene may affect the production and function of pulmonary surfactant, leading to severe lung disease in term newborns. SFTPC gene mutations may be an important risk factor for the development of common lung diseases. Because of the important roles of surfactant functions and metabolism, mutations in these genes can affect the production and function of pulmonary surfactant, leading to severe lung disease in term newborns. Further identification of related gene mutations may provide a basis for early intervention, prognosis, and genetic counseling.

## Author contributions

**Funding acquisition:** Hong-Yu Chang.

**Investigation:** Jing-Li Guo.

**Methodology:** Hong-Yu Chang.

**Resources:** Xiao-Juan Yin, Bo Cheng.

**Software:** Hai-Tao Li, Xiao-Juan Yin, Bo Cheng, Jing-Li Guo, Na Li.

**Validation:** Bo Cheng.

**Writing – original draft:** Ya-Xin Tan, Shu-Jun Li, Hai-Tao Li, Xiao-Juan Yin.

**Writing – review & editing:** Xin-Tan, Shu-Jun Li, Hai-Tao Li, Xiao-Juan Yin, Cheng-Zhong Zheng, Hong-Yu Chang.
